# Surgical procedures for nasal deformity in patients with cleft lip and palate

**DOI:** 10.20407/fmj.2025-013

**Published:** 2025-08-06

**Authors:** Yoshikazu Inoue, Hiroshi Nishioka, Maki Inukai, Makiko Yamauchi, Takayuki Okumoto

**Affiliations:** Department of Plastic and Reconstructive Surgery, Fujita Health University, School of Medicine, Toyoake, Aichi, Japan

**Keywords:** Corrective rhinoplasty, Cleft lip and palate, Septal extension graft, Nasal tip morphology, Alar cartilage

## Abstract

**Objectives:**

External nasal deformity in patients with cleft lip and palate causes both functional and aesthetic problems. Corrective rhinoplasty using a reverse U-shaped incision and suturing of the alar cartilage is not always successful. Therefore, we compared the use of a newer septal extension graft technique with the conventional suture technique to determine an effective surgical method for improving nasal tip morphology.

**Methods:**

We compared the outcomes of the conventional reverse U-shaped incision technique with the septal extension graft in 12 patients undergoing secondary cleft rhinoplasty (6 in each group). Ten plastic surgeons evaluated 6-month postoperative photographs using six criteria: nasal tip shape (frontal, lateral, and basal views), left–right asymmetry (frontal and basal views), and overall improvement. Each item was rated on a 4-point scale (1=poor, 4=excellent). Mann–Whitney U tests were used to assess statistical significance.

**Results:**

The septal extension graft group showed significantly better nasal tip morphology in the frontal and basal views (P<0.001) and in the lateral view (P=0.007). However, there were no significant differences in symmetry improvement between the two techniques for the front (P=0.685) and bottom (P=0.602) views.

**Conclusions:**

Corrective rhinoplasty using a septal extension graft can significantly improve nasal tip morphology in cleft lip cases compared with the reverse U-shaped incision and alar cartilage suturing technique. However, decreased mobility of the nasal tip was noted. Further improvement is needed in terms of patient-reported satisfaction and postoperative stability.

## Introduction

The most common congenital external surface anomaly in the cranio-maxillofacial region is cleft lip and palate, occurring in approximately 1 in 500 children in Japan. We have been treating cleft lip and palate since 1986. In 1992, we established the Cleft Lip and Palate Center at Fujita Health University Hospital, integrating the Departments of Plastic Surgery, Dental and Oral Surgery, Otolaryngology, Pediatrics, and Rehabilitation Medicine. By 2021, the cumulative number of first-time patients receiving surgery for cleft lip exceeded 2000.^1^

**Figure 1:**
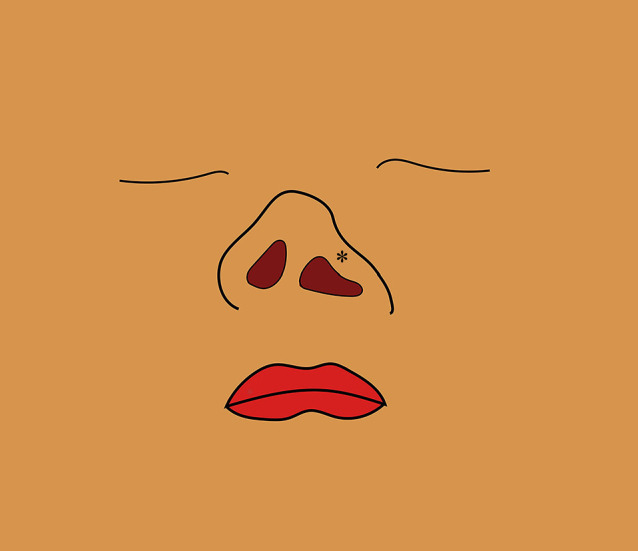
Representation of a typical external nasal deformity in a case of unilateral cleft lip and palate (left-sided cleft lip). The nasal columella is slightly collapsed on the affected side, and the ala shows a downward convex deformity (asterisk).

**Figure 2:**
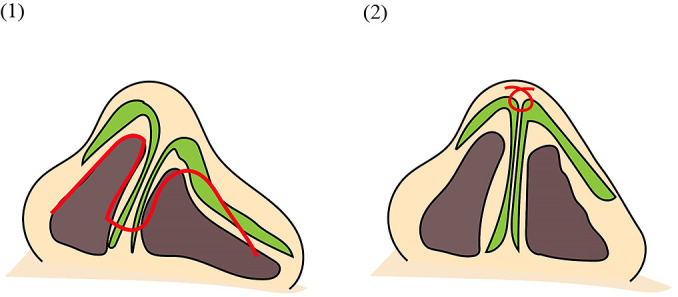
Schematic of corrective rhinoplasty for external nasal deformity in left-sided cleft lip and palate using a reverse U-shaped incision and suturing of the alar cartilages (Technique A). (1) The open approach is performed with the incision design indicated by the red line. The right nostril margin involves a rim incision, while the left nostril margin on the affected side follows a reverse U-shaped incision, extending slightly toward the dorsal nasal skin. Green indicates the alar cartilage. (2) After elevation of the skin flap using the reverse U-shaped incision, both affected and unaffected cartilage fields are dissected. Sutures (shown in red) are placed between the alar cartilages to reduce the height and morphological differences of the greater alar cartilages, which initially showed marked asymmetry. The nasal tip, which was significantly tilted, is also corrected. The skin is then sutured to complete the procedure.

One of the major challenges in the treatment of cleft lip and palate is correcting external nasal deformities.^2,3^ The nose plays a crucial role in both respiration and facial aesthetics; however, patients with cleft lip and palate often exhibit characteristic deformities of the nose.^4^ These deformities are influenced by the cleft lip and include deformity of the alar cartilages,^2,3^ with asymmetry of the left and right sides of the nose being particularly pronounced in unilateral cleft lip and palate cases (Figure 1).^5^ We have been performing secondary cleft rhinoplasty to correct nasal deformities, including those of the alar cartilages, which is one of the most difficult operations in external nose surgery.^6–8^ Previously, our standard corrective rhinoplasty method involved opening the nose with a transcolumellar incision at the base of the nasal bridge using a reverse-U incision^9^ and suturing between the alar cartilages^10^ to correct the marked asymmetry of the alar cartilages and refine nasal tip morphology (Figure 2).^11–13^ Although this method of external nose morphology correction focuses on achieving nostril symmetry,^14^ there are many cases in which this method alone does not provide significant improvement, especially in cases of short noses, for which the short nasal appearance was sometimes accentuated. Therefore, in search of a better surgical method, we changed our technique in 2010 to secondary cleft rhinoplasty that uses the septal extension graft,^15–17^ a method used in cosmetic surgery for nose tip formation. This study investigates the usefulness of the newer technique by comparing the external nasal morphology of patients who underwent secondary cleft rhinoplasty by either technique.

## Methods

We evaluated the degree of morphological improvement by comparing preoperative findings with postoperative results in 12 patients with unilateral cleft lip who underwent secondary rhinoplasty using one of two techniques: reverse U-shaped incision and suturing of the alar cartilage (Technique A) or septal extension grafting (Technique B). Each technique was applied to six patients. This study was limited to consecutive cases of incomplete unilateral cleft lip treated by the same surgeon. Bilateral cases and cases with cleft palate were excluded. This study involved 12 patients with incomplete cleft lip, aged 15 to 21 years (average age, 16.8 years), including 7 male and 5 female patients. The two techniques are described below.

### Surgical techniques

#### Technique A: Secondary cleft rhinoplasty using a reverse U-shaped incision and suturing of the alar cartilages (Figure 2)

Surgery was performed under general anesthesia following injection of 1% xylocaine hydrochloride with 1:100,000 epinephrine. All patients underwent an open approach, with a transverse incision at the base of the columella. Bilateral alar incisions included a nasolabial margin incision on the unaffected side and a reverse U-shaped incision on the affected side, with the incision line placed on the skin cephalad to the nasolabial margin. After elevating the nasal tip skin flap, dissection was carried out over the greater alar cartilages on both sides to avoid cartilage damage. Subcutaneous dissection exposed the alar cartilages. The deformed and drooping greater alar cartilage, as well as the cartilage on the unaffected side, were fully exposed and confirmed. Once dissection was complete, the bilateral alar cartilages were grasped to confirm mobility. The deformed, drooping cartilage was elevated, and an intercartilaginous suture was placed to align the dome height with that of the unaffected side. Closure was performed in stages: first, the incision at the base of the columella was sutured, followed by closure of the nasal cavity. The position of the nasal aperture margin was carefully checked to ensure that the skin of the affected nostril margin was drawn into the nasal cavity to achieve symmetry with the unaffected side.

#### Technique B: Secondary cleft rhinoplasty using a septal extension graft (Figure 3)

Surgery was also performed under general anesthesia following injection of 1% xylocaine hydrochloride with 1:100,000 epinephrine. As in Technique A, all patients underwent an open approach; however, in Technique B, a transverse inverted V-shaped incision was made slightly cephalad to the base of the columella. Bilateral infracartilaginous incisions were made in the nasal cavity of both alae. After elevating the nasal tip skin flap, dissection was performed to expose both alar cartilages (avoiding cartilage damage), extending to the lateral nasal cartilage. The deformed and drooping alar cartilage and the cartilage on the unaffected side were completely exposed and confirmed. Dissection was then performed between the right and left alar cartilages to identify the caudal end of the nasal septal cartilage. The nasal septal cartilage and nasal mucosa were dissected bilaterally and cephalad until the vertical cribriform plate was exposed. After septal dissection, an L-strut approximately 10 to 15 mm wide was preserved, and the remaining septal cartilage was harvested. The harvested cartilage was suture-fixed to the tip of the L-strut. The nasal tip shape was then constructed by suturing the bilateral alar cartilages to the extended nasal septum cartilage. After suturing the transverse incision of the nasal bridge, the nasal cavity was closed and the wound was sutured.

### Evaluation

**Figure 4:**
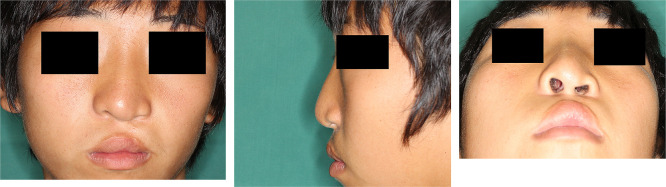
Frontal, lateral, and basal views of a case of unilateral cleft lip and palate (left-sided cleft lip).

We compared six consecutive cases from each surgical technique by assessing preoperative and 6-month postoperative photographs taken from front, side, and bottom views (Figure 4). Nasal tip morphology, asymmetry (excluding side view), and overall improvement were each scored on a 4-point scale (excellent=4, good=3, fair=2, poor=1) by 10 plastic surgeons—3 affiliated with our institution and 7 from outside institutions who had no involvement in the treatment. All statistical analyses were performed using the Mann–Whitney U test with IBM SPSS Statistics for Windows, version 29 (IBM Corp., Armonk, NY, USA). For all analyses, statistical significance was set at P<0.05. All reported P values are two-sided.

### Ethical considerations

This retrospective study was approved by the ethics committee of Fujita Health University (No. HM21-444) and conducted in accordance with the principles of the Declaration of Helsinki. Consent for participation was obtained using an opt-out method. Patient consent for publication of images has been obtained.

## Results

**Figure 5:**
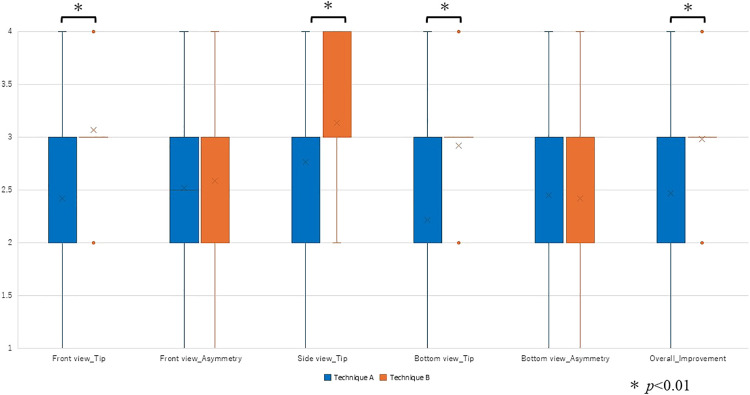
Mean and standard deviation of 10 plastic surgeons’ evaluations of revision rhinoplasty using Technique A and Technique B. Blue bars: Technique A; Orange bars: Technique B. *Statistically significant differences between techniques (P<0.01).

The 10 plastic surgeons’ evaluations of the revision rhinoplasty using the reverse U-shaped incision and suturing technique (Technique A) and the septal extension graft (Technique B) are presented in Figure 5.

In the front view, the score for nasal tip morphology improvement was significantly higher for Technique B than for Technique A (Technique A: 2 [2–3], Technique B: 3 [3–3]; P<0.001). There was no significant difference in front view asymmetry scores (Technique A: 2.5 [2–3], Technique B: 3 [2–3]; P=0.685). In the side view, the nasal tip improvement score was significantly higher for Technique B than for Technique A (Technique A: 3 [2–3], Technique B: 3 [3–4]; P=0.007). In the bottom view, nasal tip improvement was again significantly higher for Technique B (Technique A: 2 [2–3], Technique B: 3 [3–3]; P<0.001). There was no significant difference in bottom view asymmetry scores (Technique A: 2 [2–3], Technique B: 2 [2–3]; P=0.602).

The overall improvement score was significantly higher for Technique B than for Technique A (Technique A: 3 [2–3], Technique B: 3 [3–3]; P<0.001).

## Discussion

Our previous corrective rhinoplasty method used the open approach and the reverse U-shaped incision technique proposed by Tajima and Maruyama.^9^ This method, characterized by the position of the nostril margin incision, is still widely used to elevate the ptotic nostril margin cephalad, bringing it closer to the position of the unaffected side to achieve greater symmetry.^12–14^ When correcting the position of the alar cartilage, the deformed cartilage on the affected side is sutured to the cartilage on the unaffected side to align the height of the middle crus and improve nasal tip shape, thereby enhancing symmetry. However, in patients with thick skin and large sebaceous glands—features commonly seen in Asian individuals^18,19^—correction of the asymmetry between the right and left nostril margins can be achieved, but significant improvement in nasal tip morphology is often difficult. In some cases, intercartilaginous suturing may even cause additional cephalic rotation of the nasal tip, further accentuating the short nose appearance.

To overcome these issues, we adopted the septal extension graft method, which is widely used in cosmetic surgery, to create an aesthetically favorable nasal tip morphology. This technique does not prioritize nostril symmetry but instead focuses on controlling nasal tip projection and rotation. Asian individuals generally have weaker nasal cartilage than do Caucasians; when nasal apex projection is attempted solely through suturing between the alar cartilages, the framework is often compressed under the skin, and the intended nasal tip shape is not achieved. In rare cases where the medial crus of the alar cartilage is particularly strong, the nasal tip may shift cephalad when covered by the skin, also resulting in an unsatisfactory shape. To maintain nasal tip morphology and projection under the pressure of fragile cartilage and thick skin, a strong structural framework is required. Septal extension grafting—where the left and right greater alar cartilages are suture-fixed to grafted cartilage anchored to the nasal septum, a strong supporting structure—reinforces the delicate alar cartilages and helps preserve nasal tip morphology.^20^ Septal extension grafting addresses challenges in tip morphology, bulbous nose, and short nose, which are characteristic of patients with cleft lip and palate, and enables the nasal tip projection that was difficult to achieve with previous methods.

In this study, there was no significant difference between the two techniques in terms of asymmetry; however, nasal tip correction using the septal extension graft (Technique B) proved significantly superior to our previous method (Technique A) in improving the overall nasal shape. Although the degree of asymmetry improvement did not differ significantly between Techniques A and B, one reason Technique B received higher evaluations may be that the clarity of the nasal tip shape is considered an important factor in determining the aesthetic quality of the external nose. That said, Technique B is not without its challenges. Because it relies on the nasal septum, if the septum itself is fragile, there is a tendency for the nasal tip to shift postoperatively, leading to retroversion—a reduction in nasal apex projection.^21^ Additionally, the strong framework that is the main advantage of Technique B also introduces certain drawbacks. The nasal apex is normally a soft and mobile area, but in Technique B, the grafted cartilage is fixed to the caudal end of the immobile nasal septal cartilage to create the nasal tip shape, resulting in a rigid and immobile nasal tip.^22^ We have found that patients who undergo septal extension grafting often report during outpatient consultations that while the shape has improved, the nasal tip feels stiff and immobile, causing discomfort.

While improving the shape of the nose is important, we believe that the ultimate goal of correcting nasal deformity is to achieve patient satisfaction. The evaluators in this study were plastic surgeons trained to apply objective criteria for aesthetic assessment. However, there remains the possibility that subjective factors influenced their evaluations. Another limitation of this study is that the assessments were based on two-dimensional photographs rather than direct observation of the patients. Therefore, the development of more objective evaluation methods—such as those utilizing artificial intelligence—would be beneficial in future research. Additionally, future studies should incorporate patient-reported outcomes, such as the CLEFT-Q, to better assess subjective satisfaction.^23–26^

In our current clinical practice, we also perform external nose correction using columellar strut grafting and suture techniques,^27–29^ which can create a more mobile nasal tip and address some of the limitations associated with the septal extension graft technique. We believe that further studies are needed to determine which surgical approach best improves nasal appearance while also enhancing patient satisfaction.

## Figures and Tables

**Figure 3:**
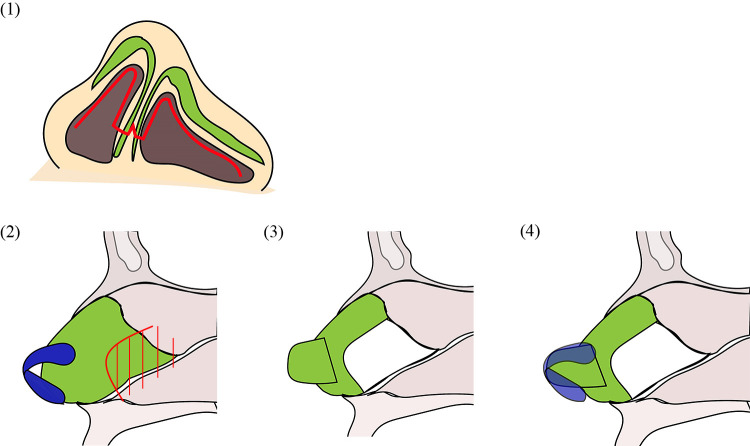
Schematic of corrective rhinoplasty for external nasal deformity in left-sided cleft lip and palate using a septal extension graft (Technique B). (1) The open approach is performed with the incision design indicated by the red line. An inverted V-shaped incision is made in the nasal columella, and the nasal cavity is accessed via infracartilaginous incisions. (2) After dissection, a portion of the nasal septum (red shaded area) is excised, leaving the L-strut intact. Blue indicates the alar cartilage, and green indicates the septal cartilage. (3) A segment of the removed nasal septum is processed and suture-fixed to the caudal end of the L-strut to extend the nasal septum. (4) The tip of the extended septal cartilage is sutured to the alar cartilage to form a new nasal tip morphology. After adjusting nasal tip rotation and projection, the skin is sutured, completing the procedure.
